# What Makes the Best Educator? A Qualitative Analysis of Emergency Medicine Resident Perspectives

**DOI:** 10.1002/aet2.70211

**Published:** 2026-07-16

**Authors:** Marshall Howell, Joshua Ginsburg, Erin Lincoln, C. Reece Brockman, D. Mark Courtney, Brian Milman

**Affiliations:** ^1^ University of Texas Southwestern Medical Center Dallas Texas USA

## Abstract

**Background:**

Effective clinical educators are essential to emergency medicine (EM) residency training, and the pace and dynamics of the emergency department require them to have a unique educational skillset. Most existing literature defining educational excellence in EM reflects educator rather than learner perspectives, and there is limited contemporary, resident‐centered work that describes the teaching practices of exceptional EM educators. This study aimed to identify EM resident‐defined practices that characterize the “best” EM educators.

**Methods:**

We performed a qualitative thematic analysis to analyze open‐ended survey responses from residents at a large, urban, 3 year EM residency program. All 78 residents were invited to complete an anonymous survey containing a single prompt to describe their best EM educator. Data were collected over 72 h in March 2025. Three investigators performed iterative open coding and applied constant comparative methods to identify themes. Postgraduate year (PGY) class was unblinded after analysis to explore patterns by training level.

**Results:**

Thirty‐eight residents responded (48.7% response rate). Five themes emerged: (1) growth orientation, (2) psychological safety, (3) shares clinical burden, (4) educational expertise, including optimal timing and setting of teaching, knowledge translation, and fostering independence; and (5) clinical expertise, including content expertise and role modeling. In a secondary descriptive analysis, PGY‐1s emphasized psychological safety, growth‐oriented feedback, and autonomy, while PGY‐2 and PGY‐3 residents prioritized approachability, timely teaching, shared workload, and clinical expertise.

**Conclusions:**

Residents identified outstanding EM educators as growth‐oriented, supportive, skilled teachers and expert clinicians, with preferences varying by training level. These themes may guide faculty development, coaching, and feedback as well as future studies on resident‐centered educator training.

## 1 Introduction

Defining what makes an excellent clinical educator has long been a challenge in medical education. While excellence in clinical teaching is widely valued, it remains a multifactorial construct that does not lend itself to a single, universal definition. This challenge is underscored by growing recognition that learning is a situated, socially‐mediated process influenced by one's relationships and environment [1, 2]. As a result, the practices that define effective educators may not translate across clinical settings and may change over years of experience of the learner.

Emergency medicine (EM) is a cognitively and operationally demanding specialty, requiring educators and learners to manage rising patient volumes, high acuity, and a wide breadth of pathology [3]. This is further complicated by the need to meet clinical metrics, manage frequent interruptions, and care for patients in atypical locations, such as chairs and hallways [4, 5]. The pace and dynamics of this environment present challenges for the application of education theory. At the same time, the ED offers uniquely rich educational opportunities through the volume of pathology and acuity, exposure to undifferentiated illness, and opportunities for close clinical supervision from attendings on shift [6]. However, being a skilled clinician does not inherently confer the skills required to teach effectively in this dynamic environment. These challenges and opportunities require that EM educators possess a unique and deliberate educational skillset [7].

The existing literature characterizing effective clinical educators largely comes from the broader field of medical education, non‐EM specialties, and other health professions [8, 9, 10, 11]. In 2008, Sutkin et al. [8] reviewed 68 articles describing characteristics of “good” clinical teachers in medical education at large, including medical knowledge, communication skills, and enthusiasm. However, most of the cited articles consisted of historical essays, surveys, and faculty perspectives, with limited attention to learners' lived experiences of clinical teaching. This pattern reflects a broader trend within the literature: educator excellence is often defined by educator‐derived frameworks, rather than by those experiencing teaching in real time. Notably, the only prior study exploring learner perspectives on EM clinical educators was from Thurgur et al. [12] in 2005 using qualitative focus groups of EM residents, medical students, and off‐service residents. While foundational in this area, that study predates major transformations in emergency medicine practice and medical education, including the widespread adoption of the electronic medical record, increased clinical throughput demands, and the evolving role of the ED within modern healthcare systems [3].

Accordingly, a contemporary, emergency medicine‐specific understanding of the teaching practices and behaviors that define excellent clinical educators is needed. This may be best approached through the perspectives of learners reflecting on their individual experiences within the unique clinical context of the ED. How residents experience the attending‐resident relationship may be central to understanding what makes a clinical educator effective. Telio et al. [13] have proposed the “educational alliance” as a framework for conceptualizing this relationship in medical education. They describe a mutual understanding of the goals of the educational relationship, agreement on how to work toward those goals, and the resident's trust in and respect for their supervisor. Critical to this concept is that the effectiveness of the educational alliance is largely determined by the learner's perception of the relationship, not the educator's. Examining what residents identify as the hallmarks of excellent clinical educators may therefore illuminate the conditions under which an effective educational alliance is established. To this end, our study employed a qualitative analytical approach using a single open‐ended, reflective survey prompt to elicit residents' descriptions of their best clinical educators. We sought to define resident‐identified practices that characterize exceptional EM educators and that may inform faculty development, coaching, and feedback strategies aligned with the realities of teaching in the modern ED.

## 2 Methods

### 2.1 Study Design

We conducted a qualitative analysis of open‐ended survey responses from emergency medicine residents. We employed qualitative thematic analysis to identify themes describing the practices of exceptional EM educators. The study was approved by the University of Texas Southwestern Medical Center Institutional Review Board (IRB) in March 2025.

### 2.2 Study Setting and Population

Data were collected from March 13 to 16, 2025 at our large, urban, 3 year academic EM residency program. We performed total population sampling, inviting all of the program's 78 residents to participate. There were 26 residents per class, with 55% male residents and 45% female. Fellows and faculty members were excluded.

### 2.3 Instrument Development

The survey instrument was developed by our team of faculty with prior qualitative research experience, including two junior faculty focused on medical education, an Assistant Program Director, the Medical Education Fellowship Director, and the Executive Vice Chair of the department. The survey was built using the REDCap platform and designed to be anonymous, collecting no personally identifying information. Respondents indicated their residency class year and gender identity. Gender identity was collected to characterize the study population and assess the representativeness of respondents, though it did not inform theme identification as all identifiers were blinded during analysis. A single, open‐ended prompt was chosen over semi‐structured interviews or focus groups to allow residents to freely define the characteristics of their best educator without the influence of predetermined categories or researcher framing. An anonymous survey format also ensured residents could respond candidly, which would have been challenging during face‐to‐face interviews or discussions with faculty, given the inherent power differential. We piloted the primary research question with five residents to ensure question clarity and strengthen response validity. Feedback from the pilot informed minor refinements to question wording and instructions. The final survey contained the following prompt:

Describe your best emergency medicine educator. What makes them stand out?

### 2.4 Study Protocol

The study was introduced and administered during resident conference on March 13, 2025. Fifteen minutes were dedicated to study introduction and responses. The IRB waived documentation of informed consent. All participants were informed that participation was anonymous, voluntary, and not incentivized. By opening the survey, they agreed to study participation. The following instructions were included on the survey form:

Please take 5 to 7 min to describe your best emergency medicine educator. Before writing, take a minute to think about the educators you admire. Try to envision a particular educator, past or present, who has impacted your career thus far. Think about what makes them stand out. Feel free to use anecdotes that highlight what makes them the best educator. Please write in sentence format, and avoid using bullets.

Residents completed the survey without research team members present and were allotted 15 min for completion. The universal survey link was sent to all residents' university email addresses and remained open for 72 h to allow participation from residents unable to attend conference. A single reminder email was sent to all residents 24 h before survey closure. All responses were exported in their unedited form to Microsoft Excel for analysis. Participants' postgraduate year (PGY) classes and gender identifiers were blinded prior to coding to reduce bias. Any responses referencing individuals were manually de‐identified prior to analysis, with names removed and gendered pronouns made neutral.

### 2.5 Data Analysis

Three investigators (MH, JG, and EL) performed open coding of the first 15 responses, identifying 101 initial codes. BM, MH, JG, and EL then reviewed and discussed the first 15 responses. Codes were consolidated to reduce overlap, resulting in an initial codebook of 45 codes.

MH and JG then coded an additional ten responses, keeping memos on code meanings and identifying gaps or redundancies. Afterward, discrepancies in coding were resolved through discussion and consensus, with BM available as an arbitrator. One code was added and two were collapsed into other codes, resulting in a final codebook of 44 codes with preliminary thematic organization. MH and JG then reapplied the new codebook to all responses. We observed thematic sufficiency after the first 25 responses, after which no new codes or concepts emerged. All 38 responses were coded using the finalized codebook to confirm the stability of the coding structure, ensure uniform application across the dataset, and identify potential negative cases. MH and JG's coding datasets were combined into a single coding document, eliminating redundancies.

All investigators then met in person to review the codebook and full survey responses. Themes and subthemes were identified using constant comparative methodology and established through group consensus. An audit trail was maintained throughout analysis, including all individual coding documents, memos, and records of codebook refinements and consensus group decisions.

After thematic analysis was complete, PGY class for each response was unblinded and used to create a table of code frequencies stratified by PGY class. These data were used to explore descriptive patterns in residents' perceptions of educators as they progress through training.

Member checking was performed with five residents after themes and subthemes were identified and again with three additional residents after the manuscript was drafted to confirm interpretive accuracy and thematic resonance.

### 2.6 Reflexivity

Throughout coding and theme identification, we remained aware of our perspectives as academic physicians at a large academic emergency medicine residency, with extensive involvement in residency education, mentoring, and curricular design. These viewpoints may have influenced more convergent data interpretation. Our group included faculty of varying seniority, from junior faculty to professor, as well as members of residency, fellowship, and departmental leadership, all of whom trained at different EM residency programs in different states. This variety of experiences allowed for more balanced qualitative interpretation. We also recognized that our shared institutional context as faculty at the program we studied may have shaped our interpretive lens, giving us insight into the program's culture but also normalizing assumptions that outside researchers may have questioned. We remained mindful of the power differential inherent to faculty researchers analyzing responses from residents they supervise, evaluate, and teach. Divergent interpretations among group members were managed through consensus discussions and a detailed audit trail. These processes were designed to surface and reconcile differences in perspective rather than suppress them.

## 3 Results

We invited a total of 78 residents to participate in the survey. We received 38 total responses (48.7%), two of which were completed after residency conference. All surveys opened were completed. Average survey completion time was 3 min and 39 s, with a range from 53 s to 8 min and 59 s. 60.5% of respondents were male and 39.5% female. PGY‐3s comprised 39.5% of respondents, PGY‐2s 31.6%, and PGY‐1s 28.9%.

We identified five themes: (1) Growth orientation, (2) Psychological safety, (3) Shares clinical burden, (4) Educational expertise, and (5) Clinical expertise. Within the theme of educational expertise, we identified three subthemes: timing and setting, knowledge translation, and fostering independence. Within the theme of clinical expertise, we identified two subthemes: content expertise and role modeling. A summary of the major themes, subthemes, and coding tree along with exemplary quotes is available in Table 1.

**TABLE 1:** Major themes and coding tree.

Themes	Subthemes	Codes	Example quotes
Growth‐oriented		Actionable feedback	“… good educators take the time at the end of shifts to provide specific, constructive, and actionable feedback.” Resident 11, PGY‐2
		Positive reinforcement	“[They] recognize that I'm new in EM and doing many things for the first time, but recognize when I do things well and gives kudos…” Resident 33, PGY‐1
		Careful criticism	“They praise good behaviors and catches in public but give criticism in private.” Resident 16, PGY‐2
		Challenges learner	“Helping students arrive at a conclusion or ‘aha’ moment by helping them stretch their thinking processes and leaving them with something impactful.” Resident 35, PGY‐3
		Growth‐oriented	“The most effective educator checks in with the student and evaluates goals and areas where the resident would like to improve and provides support on‐shift to help achieve this endpoint.” Resident 27, PGY‐3
		Normalize failure	“… pushes me to learn from my own successes and mistakes.” Resident 14, PGY‐2
Psychological safety		Psychological safety	“They create a very positive and non‐judgmental learning environment where I am able to be confident and independent, but at the same time not afraid to think out loud and express my doubts/concerns/struggles when it comes to patient care.” Resident 37, PGY‐2
		Emotional intelligence	“The best educators also get to know their residents and pick up on clues that they may be having a bad day/going through a rough time.” Resident 22, PGY‐2
Shares clinical burden		Shares clinical burden	“Will recognize when residents are struggling and offer to help.” Resident 1, PGY‐3
		Supportive	“They have your back with rude consultants or patients.” Resident 16, PGY‐2
Educational expertise	Timing/setting	Sensitive to resident workflow	“They work in teaching without disrupting flow of the department.” Resident 29, PGY‐2
		Commitment to teaching	“The best educators I've worked with are the ones that care and are passionate about education.” Resident 31, PGY‐1
		Informal teaching	“[They] find teaching opportunities during the patient's ED course.” Resident 14, PGY‐2
		Structured teaching	“They devote specific time to talk through topics, draw pictures, bring up specific articles.” Resident 22, PGY‐2
		Bedside teaching	“I have learned ways to communicate with families regarding bad news from one of my favorites. I have also learned how to use shared decision making in a way that benefits patients. One of my favorite attendings… is able to find bedside teaching opportunities out of anything.” Resident 9, PGY‐3
	Knowledge translation	Makes complex topics simple	“… breaks complicated things into simple steps/take aways that are easier to remember and apply to a shift.” Resident 24, PGY‐2
		Explains decision‐making	“[They do] not wait for me to ask all of the questions, instead providing information that I don't even think to ask, but which is always helpful.” Resident 33, PGY‐1
		Individualized approach	“This means understanding the resident's strengths and weaknesses and tailoring shifts to address weaknesses and highlight their strengths.” Resident 11, PGY‐2
		Acknowledges variability in practice styles	“Lets you make your own plans (within reason) then tells you after how [they] might have done it differently.” Resident 38, PGY‐1
	Fostering independence	Scaffolding	“The best emergency medicine educator is someone who can balance autonomy and supervision.” Resident 14, PGY‐2
		Allows autonomy	“[They] allow people to make their own decisions, and that freedom allows us to develop our own clinical practice.” Resident 38, PGY‐1
		Resident empowerment	“They go out of their way to make me feel important.” Resident 10, PGY‐2
Clinical expertise	Content expert	Content expert	“[They] constantly [update] myself and others with new data/research and [are] great at explaining things. Best of all, when asked a question [they answer] with specific detail which is great for someone learning.” Resident 6, PGY‐3
		Evidence‐based medicine	“Also someone who provides real evidence for the things they teach.” Resident 26, PGY‐1
	Role model	Role modeling	“One of my favorite EM educators has taught me by example.” Resident 23, PGY‐2
		Collaborative	“They establish a team dynamic and are not hierarchical.” ‐Resident 29, PGY‐2
		Patient‐oriented	“… consider the patient's best interest rather than solely the hard medicine.” Resident 9, PGY‐3

### 3.1 Theme 1: Growth Orientation

Residents consistently described their best educators as those who fostered clinical and personal growth through quality feedback and deliberate challenges.

They give constructive feedback and serve as a resource for career and individual development. (Resident 20, PGY‐1)

The best educators also question your choices, ultimately making you more confident in the decisions that you make. (Resident 22, PGY‐2)

By balancing positive reinforcement with careful criticism and framing mistakes as learning opportunities, these educators promoted learning while normalizing imperfection as part of the growth process.

They give praise when you do something well and help you understand why you did something wrong without being judgmental. (Resident 31, PGY‐1)

### 3.2 Theme 2: Psychological Safety

While growth orientation describes how educators actively engage learners through feedback and challenges, psychological safety refers to the comfortable learning climate that educators create.

The best teachers I have always start with trust. That lets you as a learner know that you are in a “safe place” to make mistakes. Once trust is established between learner/faculty, teaching can begin. (Resident 24, PGY‐2)

It makes it significantly more encouraging to have a teacher who you are not afraid of giving a wrong answer to. (Resident 15, PGY‐1)

They are also very approachable and are open to any question no matter how dumb. They also do not use high‐level vocabulary and instead use terms we all can understand. (Resident 10, PGY‐2)

Residents described psychological safety as a foundation for effective learning—allowing learners to engage clinical challenges with an open mind and take risks without fear of judgment.

### 3.3 Theme 3: Shares Clinical Burden

Residents highlighted the importance of educators sharing the clinical workload, particularly during busy shifts. These attendings monitored how residents were managing the patient load and intervened when necessary to protect their learning experience.

They check in with the resident throughout the shift to see where they may be struggling or need assistance (e.g., writing notes, [calling] back consults, or [seeing] a patient on their own because the resident is falling behind) and assist them where they need the help. (Resident 12, PGY‐3)

They back you up whenever you are drowning. (Resident 16, PGY‐2)

By taking on some of these tasks, these educators modeled teamwork and conveyed that resident well‐being and patient safety were shared responsibilities.

### 3.4 Theme 4: Educational Expertise

Residents described several medical education skills displayed by exceptional educators. These are divided into subthemes of timing and setting, knowledge translation, and fostering independence.

#### 3.4.1 Subtheme 1: Timing and Setting

Residents appreciated attendings who were able to teach while remaining mindful of clinical flow. These educators found teaching moments at opportune times.

[They do] a great job of teaching on shift appropriately ‐ not so little that you walk away with nothing but not so much that it impedes patient care. (Resident 38, PGY‐1)

The best educators incorporate learning pearls about cases we are seeing on shift into the time when we are at the desk documenting, etc. (Resident 2, PGY‐3)

A balance of just in time teaching while not overwhelming me on a busy shift. (Resident 18, PGY‐1)

#### 3.4.2 Subtheme 2: Knowledge Translation

The best educators were able to make complicated topics simpler and explain their clinical reasoning clearly.

One example of this that comes to mind is one of our faculty members who is an amazing teacher. They explain their approach to decompensated cardiac patients in such a simple way that even medical students in their 1st or 2nd year can follow what they are saying. (Resident 21, PGY‐2)

I also appreciate when attendings explain the thought process behind clinical decisions rather than just putting in orders quietly. (Resident 11, PGY‐2)

Residents also valued educators who adapted their instruction to the specific learner by identifying knowledge gaps and tailoring their approach to the individual's prior experience and learning preferences.

The best educators are ones that remember what it is like to be a learner, can meet the learner where they are at, and take time to learn how that learner learns. (Resident 30, PGY‐1)

They are able to recognize that learners are at different stages and adapt their teaching approach to suit the learner's style. (Resident 20, PGY‐1)

The best educators also understood that there are often acceptable approaches to providing emergency care that differ from their personal practice.

They are also flexible, don't force a certain practice pattern on me ‐ they provide their insight and experience and clinical knowledge if their practice pattern differs, but if I'm not going to hurt a patient or blow up the department they will let me continue with my proposed plan and then learn from it by seeing how it plays out. (Resident 37, PGY‐2)

#### 3.4.3 Subtheme 3: Fostering Independence

Residents felt that an educator's ability to promote autonomy while maintaining appropriate supervision was a valuable educational skill. Such educators empowered residents to act as leaders in the clinical environment.

A great educator is one that is able to remain hands off to allow the resident to practice independent medicine while also being watchful and intervene when they deem necessary to protect patient care. (Resident 12, PGY‐3)

They give you autonomy to make decisions and help you formulate a thought process for why you're making these decisions. (Resident 31, PGY‐1)

A great educator also allows the resident to lead resuscitations while quietly assisting on the side so that the ancillary staff and consultants can view the resident as the leader and main resuscitator. (Resident 12, PGY‐3)

Notably, this balance might shift depending on factors such as level of training or clinical competence.

This looks different for different levels of training and can be a big challenge. When I was first starting out as an intern, I wanted somebody who would be looking over my shoulder, filling in gaps for orders, etc. Now, as I have progressed in training, I think that the best educator is someone who can take the back seat on patient interviews, allow me to formulate my own thoughts and actions to address a patient's concerns, and then find teaching opportunities in the context of the patient's ED course… [This] pushes me to learn from my own successes and mistakes. (Resident 14, PGY‐2)

### 3.5 Theme 5: Clinical Expertise

Residents emphasized that outstanding educators were also model clinicians with deep content expertise.

#### 3.5.1 Subtheme 1: Content Expertise

Clinical credibility enhanced teaching effectiveness. Educators who could discuss evidence‐based medicine with confidence and enthusiasm while remaining humble and curious were most appreciated.

They are extremely knowledgeable on specific topics, and clearly enthusiastic about them. Simultaneously, they are aware of where their expertise ends and clear about what they do not know. (Resident 21, PGY‐2)

[They have] a wealth of clinical knowledge and still maintain a high level of enthusiastic curiosity. (Resident 23, PGY‐2)

#### 3.5.2 Subtheme 2: Role Modeling

Residents frequently described learning clinical and communication skills through observation of their educators' interactions with patients.

They are a role model clinically, and I try to learn from their bedside manner. (Resident 10, PGY‐2)

When I was an intern, [my attending] “modeled” a patient interaction for me, and it changed the way I communicate with patients even today. (Resident 17, PGY‐3)

They combine the art and science of medicine in ways that provide the highest quality of care and make working in the emergency department enjoyable and exciting. (Resident 17, PGY‐3)

### 3.6 Results by Residency Class

When code frequencies were examined descriptively by PGY level, residents of all training years placed recurring emphasis on commitment to teaching, individualized approaches to instruction, and fostering psychologically safe learning environments. PGY‐1 responses more often highlighted psychological safety, growth‐oriented feedback, and early autonomy. In contrast, PGY‐2 and PGY‐3 residents more frequently emphasized educator approachability, timely and workflow‐conscious teaching, sharing the clinical burden, resident empowerment, and clinical expertise. PGY‐2 residents in particular more commonly valued scaffolding and positive reinforcement.

## 4 Discussion

This study aimed to explore the practices and behaviors of exceptional emergency medicine educators through the lens of their residents. Using a single, open‐ended survey question, we received a wide variety of detailed and experience‐rich responses that revealed multiple themes relevant to EM educators across practice settings. The depth and authenticity of our findings may be attributed to the simplicity of the prompt, the universality of the concepts explored, and the opportunity for residents to reflect on personally meaningful experiences with minimal imposed framing. We identified five themes in their responses: growth orientation, psychological safety, sharing of clinical burden, educational expertise, and clinical expertise. Our findings echo and provide qualitative support to existing graduate medical education literature, both within and outside of emergency medicine, which emphasizes the importance of supportive learning environments and clinical and educational expertise. Several of these findings can be understood within the context of existing theoretical frameworks, underscoring the importance and applicability of foundational educational theory in the complex educational environment of the emergency department.

Our findings map coherently onto the educational alliance framework proposed by Telio et al. [13], which draws on three core components of an effective supervisor‐trainee relationship: shared goals, agreement on how to work toward those goals, and the resident's trust in and respect for the supervisor. The trust component is reflected in our data by two distinct but complementary themes: psychological safety, which captured the affective foundation residents require to engage openly as learners, and clinical expertise, with residents' trust tied to their perception of the attending's clinical credibility. The goal‐oriented components of the alliance are expressed through growth orientation and educational expertise, through which attendings calibrated the depth and timing of teaching and the degree of autonomy to each learner's stage. Sharing clinical burden extends the alliance into a dimension specific to emergency medicine, where responsiveness to the learner's needs operates both educationally and clinically, as residents value attendings who intervene during busy shifts to protect their capacity to learn. Our themes collectively describe the relational conditions under which an effective educational alliance is established, suggesting that faculty development in emergency medicine must attend to the relational dimensions of teaching, not only its technical execution.

Each of these themes can also be understood through the lens of established educational theories that help explain why they matter for learning in the emergency department.

The theme of growth orientation aligns with Dweck's growth mindset theory and highlights residents' appreciation of educators who prioritize ongoing improvement through feedback, challenge, and support. Growth mindset is the belief that attributes such as intelligence can be improved with time and effort [14]. It has been described as a key component of mastery learning, which is foundational to the competency‐based medical education model. Learners with growth mindsets value feedback and thrive in settings that foster effortful learning and challenges [15]. The breadth, pace, and uncertainty of emergency medicine ensure that trainees are frequently exposed to such clinical challenges and inevitable errors. In these situations, a fixed mindset could lead to self‐doubt or disengagement from learning, whereas a growth mindset fosters resilience and progress toward one's goals as a learner and practitioner. Educators play a vital role in the development of this mindset; graduate medical trainees have described the importance of supervisor role modeling of growth mindset for enhancing their own growth mindset [16]. Educators can therefore promote growth mindset by openly discussing personal failures and the learning that resulted. They can also provide regular feedback to trainees that encourages resilience and adaptable learning. In the fast‐paced ED, where time pressures often limit opportunities for long discussions, educators should seek opportunities for brief reflections and deliberately set aside time for thoughtful, growth‐oriented feedback to learners. Furthering the concept of growth mindset, we hope that by providing some taxonomy to domains of what learners value and expect, educators themselves may further their growth by reflecting where they might land in these domains.

A related concept described in our results is psychological safety. Popularized by business professor Amy Edmondson, psychological safety is the belief that an environment is safe for interpersonal risk‐taking without fear of negative consequences [17]. Our residents indicate that they value educators who are mindful of the trainee's psychological state and create a nonjudgmental environment built on trust. These findings support literature from other specialties demonstrating that psychological safety is important for trainees' learning and wellness [18, 19]. A scoping review of psychological safety in medical education highlighted concepts and behaviors at the organizational and team levels that promote psychological safety [20]. These included downplaying power differentials, dedicating time for relationship building, team leader enthusiasm for teaching, team leaders disclosing their own learning processes and challenges, and coaching‐oriented feedback. These behaviors can easily be implemented by educators in the emergency department by demonstrating interest in learners' lives and progress, maintaining enthusiasm for teaching throughout one's shift, embracing vulnerability and humility rather than feigning certainty, and adopting a coaching approach when appropriate.

The theme of clinical expertise highlights residents' appreciation for educators who model mastery in clinical care. This finding aligns with Social Learning Theory, which describes a process of observational learning, in which learners model and acquire new behaviors and reasoning strategies by watching others in action [21]. In the context of medical education, educators with clinical expertise serve as role models, demonstrating skills such as effective communication and clinical decision‐making that learners then emulate. BEME Guide No. 27 summarizes the evidence currently available on role modeling in medical education, emphasizing that positive role models demonstrate clinical excellence and humanistic qualities, both intentionally and incidentally [22]. This, in turn, influences learners' professionalism and career trajectories. Negative role modeling, such as cynicism and disrespect, can undermine these values, underscoring the need for awareness of and reflection on one's behaviors. For emergency medicine educators, demonstrating calm competence and compassion during stressful or frustrating ED encounters can model the balance of skill and empathy that defines the specialty.

Within the theme of educational expertise, we identified subthemes of timing and setting, knowledge translation, and fostering independence. These three teaching skills are understandably applicable to emergency medicine. Effective educators were attuned to resident workflow, identifying appropriate moments for teaching, and balancing structured and informal instruction. Given the pace, volume, and throughput needs of most EDs, it is vital that educators consider these timing and setting issues when educating. Using brief, learner‐centered teaching methods such as “One‐Minute Preceptor” or “ED STAT!” can help educators strike this balance [23]. In terms of knowledge translation, residents valued educators who could make complex topics simple, explain decision making, and acknowledge variability in practice style. By openly sharing their reasoning and acknowledging the complexity and ambiguity of many emergency department cases, educators modeled humility and contributed to the psychological safety that encourages residents to participate in the learning process without judgment. Finally, fostering independence highlighted expert educators' ability to scaffold learning (i.e., provide temporary support in completion of a learning task that would be difficult for a learner to complete independently) and encourage graduated autonomy [24, 25]. The importance of this skill is highlighted by self‐determination theory, which explains that a sense of autonomy is essential for learner motivation [26]. In the ED, educators should promote autonomy by allowing residents to make decisions while adjusting the degree of support as needed, thereby motivating residents to take ownership of their learning as they develop independent practice styles.

Few studies have explored how the teaching needs of residents evolve throughout their training. After coding all statements, we explored the frequency of codes based on year of training. In this analysis, residents' values changed as they progressed through residency. Professional identity formation (PIF), as described by Richard Cruess using Merton's words, is the dynamic process by which “medical students and residents come to think, act, and feel like a physician” [27, 28]. When we explored codes based on year of training, PGY‐1s valued safety, feedback, support, and a growth mindset. PGY‐2s valued balance: they want independence and to feel challenged, while also feeling supported and safe when they fail. PGY‐3s emphasized partnership and role modeling. This pattern aligns with previous PIF frameworks. In a 2016 article, Cruess amended Miller's pyramid to include PIF [29]. Interns are focused on the “knows,” “knows how,” and “shows how” levels of the pyramid and require feedback and support to move up to higher levels. While interns described autonomy, PGY‐2s described scaffolding. This important distinction is highlighted in a study that explored PIF in internal medicine residents, in which upper‐level residents valued the balance of autonomy and supervision. They also described the consequences of over‐supervision and lack of autonomy as well as under‐supervision and inappropriate autonomy [30]. As one of our residents explained, “A great educator is one that is able to remain hands off to allow the resident to practice independent medicine while also being watchful and intervening when they deem necessary to protect patient care.” Socialization is a critical process for PIF to progress, and our upper‐level residents valued clinical expertise. The only published emergency medicine study that investigated adaptive teaching methods based on level of trainee employed 36 h of structured observation of four medical educators in the emergency department and saw similar use of teaching methods across classes [31].

## 5 Limitations

Our study has several limitations that should be considered when interpreting the findings. First, data were collected from residents from a single, large, urban residency program, and the practices of ideal educators may differ across institutions, training models, and practice environments. Resident perceptions of ideal educators may also reflect the ideals and culture of their training program, making it difficult to distinguish broadly generalizable preferences from those that are formed by local standards. Our program is a 3 year residency, and perspectives from PGY‐4 residents at a 4 year program may have revealed additional or different insights. While many themes were observed across training levels, differences in trends between interns and more experienced residents may have been more pronounced with additional training experience. Furthermore, we had nearly twice as many male as female respondents, which is out of proportion to the program's overall gender demographic. This discrepancy may have influenced the representation of learning preferences and ideal educator descriptions.

The use of a single written prompt, while it encouraged individual interpretation, may not have captured the full breadth of practices residents associate with their best educators or the depth of reflection that interviews or focus group dialogue might produce. Responses may have been subject to recency bias, with residents emphasizing attributes of educators encountered recently, rather than those who most influenced their development overall. Responses were collected via online survey, and we attempted to reduce non‐response bias by sending the survey link to all residents, including those not in attendance at conference. The response rate of 48.7% reflects our use of total population sampling across 78 residents, including those on clinical shifts or vacation who could not attend conference. Of residents present at conference, 90% completed the survey, with two additional responses received afterward. The voluntary nature of the survey and discrepancies in length of written responses may have introduced response bias by amplifying the viewpoints of those who responded and had more detailed responses.

Finally, the stratified analysis by PGY level was exploratory in nature. Code frequencies were examined only after themes for the primary qualitative analysis had been established, which may have introduced confirmation bias for the stratified analysis. PGY‐stratified findings are presented as descriptive, rather than statistical comparisons. These observations are intended to generate hypotheses for future studies, rather than to draw definitive conclusions about developmental differences across residency training.

## 6 Conclusions

Emergency medicine residents consistently described the practices of their best educators with themes of growth orientation, psychological safety, sharing of clinical burden, educational expertise, and clinical expertise, with a focus on commitment to teaching. While preferences varied descriptively by training level, the primary themes reflect core educational practices of the best EM educators that align with residents' learning needs and established frameworks in medical education.

Areas for future research include the application of these resident‐defined practices of the best EM educators to faculty continuing education, coaching, and feedback initiatives, deeper investigation into differences in themes between residency classes, as well as a postgraduate survey of residents who have entered independent practice.

## Author Contributions

**Marshall Howell:** conceptualization, investigation, writing – original draft, writing – review and editing, validation, methodology, formal analysis, project administration, data curation, supervision. **D. Mark Courtney:** conceptualization, investigation, writing – review and editing, supervision. **Brian Milman:** conceptualization, investigation, writing – original draft, writing – review and editing, validation, methodology, formal analysis, project administration, supervision, data curation. **Joshua Ginsburg:** conceptualization, investigation, writing – original draft, writing – review and editing, validation, methodology, formal analysis, project administration, data curation. **Erin Lincoln:** conceptualization, investigation, writing – original draft, writing – review and editing, methodology, formal analysis. **C. Reece Brockman II:** writing – original draft, writing – review and editing.

## Funding

The authors have nothing to report.

## Conflicts of Interest

The authors declare no conflicts of interest.

## Data Availability

The data that support the findings of this study are available from the corresponding author upon reasonable request.
